# The Association between Sweet Taste Function, Anthropometry, and Dietary Intake in Adults

**DOI:** 10.3390/nu8040241

**Published:** 2016-04-23

**Authors:** Julia Y. Q. Low, Kathleen E. Lacy, Robert McBride, Russell S. J. Keast

**Affiliations:** 1 Centre for Advanced Sensory Science, School of Exercise and Nutrition Sciences, Deakin University, Burwood, Victoria 3125, Australia; j.low@deakin.edu.au (J.Y.Q.L.); metametrics@hotmail.com (R.M.); 2 Institute for Physical Activity and Nutrition Research, School of Exercise and Nutrition Sciences, Deakin University, Burwood, Victoria 3125, Australia; katie.lacy@deakin.edu.au

**Keywords:** sweet taste function, sweet taste, dietary intake, high intensity sweeteners, sugar, BMI, sweet taste intensity

## Abstract

Variation in ability to detect, recognize, and perceive sweetness may influence food consumption, and eventually chronic nutrition-related conditions such as overweight and obesity. The aim of this study was to investigate the associations between sweet taste function, anthropometry, and dietary intake in adults. Participants’ (*n* = 60; mean age in years = 26, SD = ±7.8) sweet taste function for a range of sweeteners (glucose, fructose, sucrose, sucralose, erythritol, and Rebaudioside A) was assessed by measuring detection and recognition thresholds and sweetness intensity. Height, weight, and waist circumference were also measured, and participants also completed a Food Frequency Questionnaire. There was large inter-individual variation in detection, recognition and sweetness intensity measures. Pearson’s correlation coefficient revealed no robust correlations between measures of sweet taste function, anthropometry, and dietary intake, with the exception of suprathreshold intensity, which was moderately correlated with total energy intake (*r* = 0.23–0.40). One-way analysis of variance revealed no significant differences between the most and least sensitive participants in terms of BMI, waist circumference, and dietary intake for all measures of sweet taste function and sweeteners (all *p* > 0.01). When stratified into BMI categories, there were no significant differences in any measure of sweet taste function between the normal weight and overweight/obese participants (all *p* > 0.01). Results show that that sweet taste function is not associated with anthropometry and sweetness intensity measures are the most appropriate measure when assessing links between sweet taste and food consumption.

## 1. Introduction

Increased energy intake, in particular greater intakes of sweet, energy-dense food, is thought to be one of the major contributors to the global rise in being overweight and obese [1,2]. For example, excessive consumption of sugar-sweetened beverages has been linked to the rising rates of obesity worldwide [3,4,5]. The continued increase in the worldwide prevalence of nutrition-related chronic illness such as obesity necessitates an increased understanding of the drivers of food intake [1,6,7,8].

The sense of taste, one of the traditional five senses (sight, hearing, taste, smell and touch), is activated when nutrients or other chemical compounds stimulate specialised taste receptor cells within the oral cavity [9]. From an evolutionary perspective our taste system functions as a gatekeeper to ingestion ensuring that we consume essential nutrients for survival and functioning, while rejecting potentially harmful or toxic foods [10]. However, research on sweetness, energy intake, and body mass index (BMI) is controversial (see reviews by [11,12,13]).

The role of taste sensitivity in promoting intake of specific foods or ingredients associated with obesity has long been an area of interest, but with mixed experimental support [14,15,16,17,18,19,20,21,22,23]. In regards to sweet taste, whether or not environmental influences such as habitual diet can alter sweet taste sensitivity or *vice versa* is still unclear. Some have reported an inverse association between BMI and sweet taste sensitivity (decreases in BMI were associated with increased sweet sensitivity) [22,24], whereas a large body of evidence indicates that there is no significant association between BMI and sweet taste function [21,25,26,27,28,29,30,31,32]. Similar complexities were also found in studies investigating the link between sweetness liking and BMI, where most data showed no link between hedonics of sweetness and body size [26,29,31,33,34,35,36]. A confounding factor in this area is that high-intensity sweeteners generally contain only negligible amounts of kilojoules thereby decoupling sweetness from energy value [37].

Discrepancies between studies may be attributed to differences in the types of sugar and/or psychophysical techniques used to measure sweet taste function [38] as research has shown that no single psychophysical measure reflects taste function in totality [39]. There are three perceptual dimensions of sweet taste function, namely detection threshold, recognition threshold, and suprathreshold intensity, each of which is independent of the other [39,40]. The present study aims to investigate associations between the three common measures of sweet taste function, anthropometry and dietary intake among adults using multiple sweeteners.

## 2. Materials and Methods

### 2.1. Study Design

This study comprised three measures of taste perception routinely used in chemosensory research: (1) detection threshold (DT); (2) recognition threshold (RT); and (3) suprathreshold intensity (ST). These measures were determined for each participant, and each sweetener, over a total of 16 sessions (two sessions per day separated by a minimum of 1 h for eight non-consecutive days). All measurements were collected in duplicate. If there were more than three concentration steps between the duplicate measures, participants attended another session to complete the assessment. Demographic information was also collected, including sex, age, height, weight, and waist circumference. Body mass index (BMI, kg/m^2^) was calculated from the height and weight measurements. DT, RT, and ST intensity tasks were conducted in computerised, partitioned sensory booths in the Centre for Advanced Sensory Science using Compusense Five Software Version 5.2 (Compusense Inc., Guelph, ON, Canada). Filtered deionised water was used as an oral rinsing agent. Participants were instructed to rinse their mouths with filtered deionised water for five seconds before beginning each task and between each sample set. To eliminate any visual and olfactory input, all testing sessions were conducted under red lighting, and participants were asked to wear nose clips during testing. All solutions were served at room temperature, with a three digit code allocated to each sample.

### 2.2. Participants

Sixty participants (28 male, 32 female), 18–52 years of age (mean age in years = 26, SD = ±7.8), were recruited from locations adjacent to the Deakin University, Melbourne campus, Australia. Subject exclusion criteria were individuals who are: (1) smokers; (2) pregnant or lactating; (3) taking any prescription medication that may interfere with their ability to taste; and (4) with a history of food allergies that may interfere with the study. Participants were asked to refrain from eating, drinking (except room temperature water), brushing teeth, and chewing gum for 1 h prior to testing. All participants gave written informed consent and were compensated for their time. This study was conducted according to the institutional review board regulations of Deakin University (DUREC 2013-156). The experimental protocol was also registered under the Australian New Zealand Clinical Trials Registry (ACTRN12613000701729), www.anzctr.org.au.

### 2.3. Participant Training

Prior to using the general Labeled Magnitude Scale (gLMS) to rate taste intensity, participants were trained using the standard protocol outlined by Green *et al*. (1993, 1996) except the top of the scale was described as the “strongest imaginable sensation of any kind” [41]. The 100-point scale comprised the following adjectives: “no sensation” = 0, “barely detectable” = 1.5, “weak” = 6, “moderate” =17, “strong” = 35, “very strong” = 52, and “strongest imaginable” = 100 [41]. Only scales with adjectives were presented to participants (no equivalent numbers, although numerical data was extracted from the scale for data analysis; [39]). During the training session, participants were asked to rate the intensity of the perceived sensation relative to a remembered or imagined sensation. Participants were required to rate a list of seven remembered or imagined sensations, such as the warmth of lukewarm water, the pain from biting of the tongue, and the sweetness of fairy floss (known as cotton candy in the USA, or candy floss in the UK).

### 2.4. Stimuli

Both caloric (glucose monohydrate, fructose, sucrose, erythritol) and high-intensity sweeteners (HIS) (artificial sweetener: sucralose; natural high intensity sweetener: Rebaudioside A) were used to investigate sweet taste (for details of stimuli see Table 1). On the morning of testing, solutions were prepared with filtered deionised water (Cuno Filter Systems FS117S, Meriden, CT, USA) and stored in glass beakers at room temperature (20 °C ± 1 °C).

### 2.5. Detection and Recognition Threshold Determination for Sweet Taste

Detailed in Table 1 are the ranges of chemical concentrations used to assess DT and RT for sweet taste. The concentration series for sucrose was adapted from ISO3972 [42]. The concentration series for the remaining sweeteners were prepared with successive 0.25 log dilution steps. Initial starting concentrations were determined through informal bench top testing, based on modified findings of matching sweetness intensity ratios published in Keast *et al*. [43]. DTs for each of the sweeteners were determined using ascending series 3-Alternate Forced Choice methodology [19,44], in which the participants were provided with three 25 mL samples: two controls (filtered deionised water solutions) and one containing sweetener per set, in ascending order from the lowest to the highest concentration. DT was defined as the concentration of sweetener required for a participant to correctly identify the sweetened sample as “odd” from the two water control samples in three consecutive sample sets at one concentration level [43]. RTs for each of the sweeteners were measured using a whole-mouth, sip-and-spit procedure [45]. Each participant received a single 15 mL sample presented in a medicine cup, in ascending order starting from his or her DT concentration level. Participants were asked to identify the quality of the taste after holding the sample in their mouth for at least five seconds. Response options included “sweet”, “sour”, “bitter”, “salty”, “umami” or “unknown taste”. Participants tasted each sample once, in ascending concentration order, until they identified the target taste quality “sweet” for all of the sweeteners [45]. RT was defined as the concentration at which they were able to recognize the correct taste quality three times consecutively. To prevent participants from learning the purpose of the task, participants were told that the purpose of this experiment was to investigate if they were able to detect any other potential taste qualities before the final “sweet” perception. They were also given examples of how some people were able to detect other taste qualities such as bitterness when tasting HIS. Thus, we found that this strategy encouraged participants to attempt recognition (not only sweet) prior to concentrations associated with probabilistic recognition (*i.e.*, the concentrations at which participants were able to recognize quality imperfectly at above chance level) [45]. At the end of the final visit, participants were debriefed about the experiment, and none were aware that the purpose of this task was a sham.

### 2.6. Suprathreshold Intensity Ratings for Sweeteners

Three concentrations (blank (control), weak, moderate, and strong) were prepared to determine perceived suprathreshold intensity for each sweetener (Table 2). These concentrations were derived through informal bench top testing (ascending taste intensity). The concentrations for each sweet stimulus ranged from “weak” to “strong” on the gLMS. These samples were presented to participants in randomised order to taste.

### 2.7. Standardisation of gLMS Usage with Weight Ratings

To standardise gLMS usage within participants, a modified version of Delwiche *et al*. [46] was adapted for this study. To control for idiosyncratic scale usage, participants were asked to rate the heaviness of six visually identical weights (*i.e.*, sand and stone filled opaque bottles completely wrapped with aluminium foils of weights 53, 251, 499, 724, 897, and 1127 g). Participants were asked to hold out their non-dominant hand palm up, while the experimenter placed the weighted bottle on the palm of the hand. Participants were instructed to use the gLMS to rate the heaviness of each weight.

Significant correlations were found between the overall mean prototypical ratings and overall mean heaviness ratings (*r* = 0.28, *p* < 0.05) (see Table 3 for concentration of prototypical tastants used for determination of taste intensity perception). As individual ratings for taste intensity and the heaviness of the bottles were assumed to be unrelated, the significant correlation indicated that the gLMS ratings were prone to individual scale-use bias and required standardisation across participants [39,40,46]. To determine a personal standardisation factor, the grand mean for heaviness across weight levels and participants was divided by each participant’s average intensity for heaviness [40]. Each individual’s intensity ratings were multiplied by his or her personal standardisation factor for scale-use bias [40,46].

### 2.8. Anthropometry

All participants were asked to remove shoes and heavy clothing to ensure accurate measurements. All anthropometry measurements were measured first thing during the initial and final visits after 1 h fast (food only). Participants’ body weight was measured to the nearest 0.1 kg using a segmental body composition analyser (TBF-300A) (Tanita Corporation, Tokyo, Japan). Participants’ height was measured to the nearest 0.1 cm using a portable stadiometer (Seca213) (Seca, Eilbek, Germany). All measurements were repeated twice to ensure accuracy. An average of measurements for both height and weight were used to calculate BMI (weight in kg/m^2^) and determine weight status (*i.e.*, normal weight or overweight/obese). Weight statuses were defined under World Health Organisation BMI classification [47]. Using methods outlined by the Australian Heart Foundation [48], waist circumference was also measured. Waist circumference was measured twice to the nearest 0.1 cm using an ergonomic circumference measurement tape (Seca201) (Seca, Eilbek, Germany). An average of waist circumference measurements was calculated and used for analysis.

### 2.9. Dietary Intake

The Cancer Council of Victoria validated Food Frequency Questionnaire (FFQ) was used to measure each participant’s habitual pattern of food intake [49]. Participants were required to indicate, on average, how many times in the previous year they consumed a number of food and beverage items (80 items) across four categories (*i.e.*, cereal foods, sweets, and snacks; dairy products, meats and fish; fruit; and vegetables). They were also required to indicate the portion size that they normally consumed. Participants were asked to complete the FFQ within a month from their first visit.

In addition to the FFQ, participants were asked how often they consumed foods and/or beverages sweetened with high-intensity sweeteners (artificial sweeteners, natural high-intensity sweeteners) by selecting an appropriate response category from a list of “more than once a day”, “once per day”, “three to six times per week”, “once or twice per week”, “one to three times per month”, “once per month or less”, or “never” [50]. Participants were given examples of commercial products that were sweetened with high-intensity sweeteners (both artificial and natural high intensity sweetened) such as low energy carbonated drinks, confectionary and dairy products. Participants were also instructed to include high-intensity sweeteners consumed with tea and coffee.

### 2.10. Statistical Analysis

SPSS Version 20.0 software (SPSS, Chicago, IL, USA) was used for the statistical analysis of the data. Data are expressed as means ± standard error of mean (SEM). Descriptive statistics were employed to describe demographic information, sweet taste thresholds and perceived sweetness intensity, dietary intake, and high-intensity sweeteners consumption. Sweet taste thresholds and sweet suprathreshold intensity ratings for prototypical tastants and sweeteners were determined as the arithmetic mean of the duplicate measures. For sweet intensity ratings, a geometric mean score of the three ratings (weak, moderate, and strong) for all sweeteners was calculated. Over and under reporters for dietary intake were checked for out of range values for energy intake and cases with outlying values (>2 SD from mean energy intake per day) were removed from further dietary analyses [51]. However for BMI and waist circumference, all participants were included in the analysis.

Sweet taste function for each sweetener was treated as a grouping variable (quartiles) with participants categorized as hyper-sensitive (1/4), normal sensitive (2/4–3/4), and hypo-sensitive (4/4) to explore differences between continuous (BMI, waist circumference, dietary intake, habitual energy intake, and macronutrient intakes) and categorical (high-intensity sweeteners consumption) variables. Sweet taste function for each sweetener was grouped into quartiles to allow comparison of most and least sensitive groupings. A one-way ANOVA with a Tukey *post hoc* analysis was used to detect differences in habitual energy intake, BMI, and waist circumference between hyper-sensitive and hypo-sensitive participants (lower and higher quartile groups). Chi-square test was used to detect differences in consumption frequency of high-intensity sweeteners between sweet taste function groups. A one-way ANOVA with a Tukey *post hoc* analysis was used to detect differences in diet between hyper-sensitive and hypo-sensitive participants, with macronutrient composition (*i.e.*, percent energy from total sugar, starch, and carbohydrate) as a dependent variable and sweet taste function as the independent variable. A one-way ANOVA with a Tukey *post hoc* analysis was also used to assess differences in terms of weight status between hyper- and hypo-sensitive participants (*i.e.*, BMI and waist circumference as a dependent variable and sweet taste function as the independent variable). Pearson’s product-moment coefficients correlations were conducted to also analyse the relationship between sweet taste function and BMI, waist circumference, and dietary intake. Independent *t*-tests were used to analyse differences in terms of sex between sweet taste function, anthropometry, and dietary intake. Significance was accepted at *p* < 0.01 to reduce the possibility of making a type I error due to multiple tests being conducted. The *p*-values were not adjusted for multiple comparisons by the application of Bonferroni or other equivalent method, as these approaches can be overly conservative (increasing risk of type II error) and can potentially mask important findings [52,53].

## 3. Results

Baseline characteristics of all participants are detailed in Table 4.

### 3.1. Sweet Taste Function of Sweet Tastants

Of the 60 participants, *n* = 15 (25%) were asked to complete an additional session due to variability in measurements. There were no significant differences in sweet taste function between male and female participants, therefore, the data are presented together (all *p* > 0.01). The DT and RT means, standard error, and range for all sweeteners are presented in Table 5. The geometric mean, standard error, and range of intensity ratings of all sweeteners measured are presented in Table 6.

### 3.2. Sweet Taste Function and Anthropometry

No significant associations were identified between any measures of sweet taste function (DT, RT, and intensity) with BMI, and waist circumference for all sweeteners tested (all *p* > 0.01). Similarly, when grouped into quartiles, there were no significant differences between the hyper- and hypo-sensitive participants according to their sweet taste function (all sweeteners) for BMI and waist circumference (all *p* > 0.01). When stratified into BMI categories, there were no significant differences in any measure of sweet taste function between the normal weight and overweight/obese participants (all *p* > 0.01).

### 3.3. Sweet Taste Function and Dietary Intake

Participants (*n* = 4) were identified as over-reporters of energy intake (>2 SD ± 10,800.2 kJ). When stratified into body sizes, overweight/obese participants had a significantly greater mean total energy intake (13,847.6 (SEM 1264.8) kJ) in comparison to normal weight participants (9235.3 (SEM 706.4) kJ). There were no other significant differences in dietary intake between normal weight and overweight/obese participants (all *p* > 0.01).

### 3.4. Sweet Taste Function and Energy and Macronutrient Intakes

The mean and standard error for energy and macronutrient intakes (in percentages of energy intake) are presented in Table 7. No correlations were observed between DT and RT, with mean total energy intake, percent energy from total fat, protein, carbohydrate, sugar, starch, and fibre (all *p* > 0.01). Significant correlations were observed between mean total energy intake and sweetness intensity ratings for Rebaudioside A (*r* = 0.40, *p* < 0.01) and sucralose (*r* = 0.36, *p* < 0.01). However, no significant correlations were identified for mean total energy intake and sweetness intensity ratings for glucose (*r* = 0.30, *p* = 0.02), fructose (*r* = 0.30, *p* = 0.03), sucrose (*r* = 0.26, *p* = 0.05), and erythritol (*r* = 0.23, *p* = 0.09). No correlations were observed between sweetness intensity for all sweeteners and percent energy from total fat, protein, carbohydrate, sugar, starch, and fibre (all *p* > 0.01). When grouped into quartiles, Tukey *post hoc* analyses revealed no significant differences between hyper- and hypo-sensitive participants (lower and higher quartiles) according to their sweet taste measures and the macronutrients investigated (all *p* > 0.01). No robust differences were observed between male and female participants in terms of the associations between sweetness intensity and energy intake (unreported).

### 3.5. Sweet Taste Function and Consumption of Added Sugar and Specific Sugar-Sweetened Foods

The mean and standard error for consumption of added sugar and specific sugar-sweetened foods (sweet biscuits, cakes, flavoured milk, fruit spreads, ice-cream, chocolate, bread, and breakfast cereals) in grams are presented in Table 8. No robust associations between measures of sweet taste function, sugar, and specific sugar-sweetened foods were observed. When grouped into quartiles (DT, RT, sweetness intensity), there were no significant differences between the hyper- and hypo-sensitive participants for all sweeteners in terms of consumption of added sugar and specific sugar-sweetened foods (all *p* > 0.01). There were no significant differences in terms of intake of added sugar and specific sugar-sweetened foods between overweight/obese subjects and normal-weight participants (all *p* > 0.01).

### 3.6. Sweet Taste Function and Consumption of High-Intensity Sweeteners

Most participants did not consume artificial sweeteners (76.8%, *n* = 43) or natural HIS (96.4%, *n* = 54) in foods and beverages. Of the participants who did consume artificially sweetened foods and beverages, 16.1% (*n* = 9) reported consuming them, on average, three to six times per week, and 7.1% (*n* = 4) reported consuming them at least once per day. There were no significant differences between measures of sweet taste function and frequency of consumption of artificially sweetened foods and beverages (all *p* > 0.01). There were no significant differences in terms of frequency of consumption of artificial and natural HIS between normal weight and overweight/obese participants (all *p* > 0.01).

## 4. Discussion

To our understanding, this was the first comprehensive study to investigate if multiple measures of sweet taste function using a range of sweeteners were related to anthropometry measurements or dietary intake. Overall sweet taste function was not associated with anthropometry measurements or dietary intake, except for mean total energy intake, where moderate correlations were found between sweetness intensity for HIS, Rebaudioside A and sucralose. A trend towards significance was also found between total energy intake and sweetness intensity for all the other sweeteners as well.

Overall, the current findings indicate that DT and RT are not associated with dietary intake and body weight. The role of sweet taste in promoting intake of foods or ingredients associated with obesity has long been an area of interest, but with mixed experimental support [38]. The findings provide no experimental evidence for a relationship between measures of sweet taste function and body size (*i.e.*, [21,25,26,27,28,29,30,31,32]) and between most measures of sweet taste function and dietary intake [21].

The moderate and near significant relationships observed between energy intake and sweetness intensity of two HIS, suggests that intensity ratings are more appropriate when assessing sweet taste associations with energy intake in comparison to sweetness DT and RTs. Similarly, one recent small-randomised controlled trial study looked at the effect of reducing intake of simple sugars on DT and ST intensity for sucrose [54]. In this study, 13 participants completed a three-month low-sugar diet and 16 participants (control group) remained on their normal diet [54]. No significant changes in sweet DT were found during the intervention in both groups, but the low-sugar diet group rated sweet puddings as more intense in months two and three of the intervention [54]. A similar, but weaker effect on rated sweetness intensity for flavoured beverages was also found [54]. This supports the general school of thought on psychophysical technique comparisons [38,54,55,56], where taste thresholds have previously been found to have limited utility in predicting experiences in the real world as threshold measures do not depict the dynamic range of sensory function. Thus, the comparison of the ability of an individual to detect and recognize sweetness from a very small amount of sugar/sweet stimuli may not be as relevant in terms of understanding food behaviour, when most of the sweet and high-energy foods are within the sweetness intensity perception range [38,55]. As absolute taste threshold measures are time consuming to complete, sweetness perceptions may seem to be a more time efficient method to assess relationships between individual sweet taste function and energy intake.

There are possible explanations for the lack of association between sweetness perception and anthropometry. First, one could argue that perception of tastant solutions in a laboratory setting bears little relevance of actual intake of real food in everyday life [57]. The impact taste perception has especially among adults is much less understood [58]. For example, sweet liking and aversions are not always direct predictors of intake, and they do not always associate sweetness with liking [57]. Therefore, it has proved difficult to link adult taste liking with body sizes and diet, whether in a laboratory setting or in the real world [58]. In truth, the proportion of sugar in an individual’s diet can be driven by many factors ranging from molecular biology to socio-economical factors such as education level and income [58,59,60,61]. Second, as obesity has been associated with diets containing high levels of both fat and sugar [62,63,64], sweet food choices may be influenced to some extent by the participant’s sensitivity and/or preference for fatty foods [25,28,38,58,64,65]. In addition, it is possible to have a diet that is considered to include many high-energy sweet foods, but without the energy coming from sweetness (e.g., energy bars/muesli bars sweetened with HIS, baked goods sweetened with HIS, *etc*.). As we only measured frequency of consumption of HIS, we do not have data on the quantity and the types of foods that participants consumed that were sweetened with HIS.

There are some limitations that need to be considered when considering the results. Food Frequency Questionnaires may not accurately reflect diet, and are prone to under and over-reporting. Moreover, there are considerable sweetener options currently available and while the authors used multiple sweeteners in this study there were other sweeteners available that were not used which may have altered the results. The unequal distribution between males and females in the BMI groups may also be a limitation of this study.

## 5. Conclusions

An individual’s ability to detect and recognize a range of sweeteners did not play a role in sweet food consumption, HIS consumption, or more generally the dietary intake of adults. Sweetness intensity from two HIS was associated with energy intake indicating that intensity measures might be more appropriate when assessing associations with total energy intake. There were no associations found between anthropometry measurements and sweet taste function across a range of sweeteners. Supporting this there were no differences in sweet taste function between lean and overweight/obese participants. Although all measures of sweet taste function differed between individuals for all sweeteners, oral sweet taste sensitivity does not appear to have any robust influence on anthropometry measurements and dietary intake.

## Figures and Tables

**Table 1:** Concentrations (milli Molar) of glucose monohydrate, fructose, sucrose, sucralose, erythritol, and Rebaudioside A to determine detection and recognition taste thresholds.

Reference Substance	Sample Concentration (mM)											
	1	2	3	4	5	6	7	8	9	10	11	12
Glucose Monohydrate	1.0	1.6	2.7	4.5	7.4	12.1	20.0	33.0	54.5	89.9	148.3	244.7
Fructose	0.6	1.0	1.6	2.6	4.4	7.2	11.8	19.6	32.3	53.4	88.1	145.4
Sucrose	0.4	0.6	1.0	1.6	2.7	4.5	7.5	12.6	21.0	35.0	57.8	95.4
Sucralose	0.0005	0.0008	0.0014	0.0023	0.0038	0.0063	0.010	0.017	0.03	0.05	0.08	0.13
Erythritol	1.5	2.4	4.0	6.6	10.9	18.0	29.6	48.9	80.7	133.0	220.0	363.0
Rebaudioside A	0.001	0.002	0.003	0.004	0.007	0.012	0.02	0.03	0.05	0.09	0.15	0.25

The concentration series for sucrose was adapted from ISO3972 [42]. The concentration series for glucose monohydrate, fructose, sucralose, erythritol, and Rebaudioside A were prepared with successive 0.25 log dilution steps. Reference chemical details: glucose monohydrate (The Melbourne Food Depot, Melbourne, Australia); fructose (The Melbourne Food Depot, Melbourne, Australia); sucrose (CSR, Yarraville, Australia); sucralose (The Melbourne Food Depot, Melbourne, Australia); erythritol (AuSweet, Melbourne, Australia); and Rebaudioside A (AuSweet, Melbourne, Australia).

**Table 2:** Concentrations (milli Molar) of glucose monohydrate, fructose, sucrose, sucralose, erythritol, and Rebaudioside A to determine taste intensity perception.

Reference Substance	Sample Concentrations (mM)		
	Weak	Moderate	Strong
Glucose monohydrate	240	480	960
Fructose	140	280	560
Sucrose	100	200	400
Sucralose	0.14	0.28	0.56
Erythritol	400	800	1600
Rebaudioside A	0.27	0.54	1.08

**Table 3:** Concentrations (milli Molar) of tastants used for determination of taste intensity perception for prototypical tastants.

Taste Quality	Reference Substance	Sample Concentrations (mM)		
		Weak	Moderate	Strong
Sweet	Sucrose	100	200	400
Salty	Sodium chloride	100	200	400
Bitter	Caffeine	1.0	2.0	4.0
Sour	Citric acid	1.0	3.0	7.0
Umami	Monosodium glutamate	3.0	6.0	12.0

The concentration series were adapted from Webb *et al*. [39]. Reference chemical details: sucrose (CSR, Yarraville, Australia); sodium chloride (Saxa, Premier Foods Inc, Seven Hills, Australia); citric acid (Ward McKenzie Private Limited, Altona, Australia); caffeine (Sigma Aldrich, Steinham, Germany); and monosodium glutamate (Ajinomoto Cooperation, Tokyo, Japan).

**Table 4:** Baseline characteristics of study participants * (Mean values and standard errors).

	All (*n* = 60)		Normal Weight (*n* = 38) ^1^		Overweight/Obese (*n* = 22) ^1^	
	Mean	SEM	Mean	SEM	Mean	SEM
Age (years)	26.5	1.0	26.5	1.4	26.3	1.3
Height (cm)	168.1	1.3	166.1	1.6	171.7	1.8
Weight (kg)	68.0	1.8	60.5	1.6	81.0	2.4
BMI (kg/m^2^) ^1^	23.9	0.4	22.0	0.3	27.3	0.4
BMI range (kg/m^2^) ^1^	18.5–32.9		18.5–24.9		25.1–32.9	
Waist circumference (cm)	80.5	1.6	73.3	0.9	93.0	2.3
Waist circumference range (cm)	59.0–112.0		59.0–85.5		73.0–112.0	

^1^ Normal weight, BMI = 18.5–24.9 kg/m^2^; overweight, BMI = 25–29.9 kg/m^2^; obese, BMI ≥ 30 kg/m^2^ [47].

**Table 5:** Taste thresholds (mM) of sweet tastants presented as mean, standard error, and range.

	Detection Threshold (*n* = 60)		Recognition Threshold (*n* = 60)	
	Mean ± SEM	Range	Mean ± SEM	Range
Glucose	17.2 ± 2.5	1.0–89.9	35.2 ± 4.0	2.7–148.3
Fructose	9.3 ± 1.4	0.6–53.4	19.7 ± 2.1	1.6–88.1
Sucrose	5.5 ± 0.8	0.4–21.0	11.7 ± 1.2	1.0–57.0
Sucralose	0.013 ± 0.002	0.0005–0.09	0.02 ± 0.002	0.0014–0.048
Erythritol	23.0 ± 3.0	1.5–80.7	44.7 ± 4.2	2.4–133.2
Rebaudioside A	0.02 ± 0.002	0.001–0.05	0.03 ± 0.003	0.002–0.09

**Table 6:** Geometric mean (gLMS), standard error, and range sweetness intensity ratings of sweet tastants.

	All (*n* = 60)	
	Mean ± SEM	Range
Glucose	15.8 ± 1.1	4.9–39.6
Fructose	15.4 ± 1.0	4.9–31.5
Sucrose	15.5 ± 1.1	4.1–44.8
Sucralose	11.6 ± 0.7	2.8–28.5
Erythritol	16.4 ± 1.2	4.7–44.8
Rebaudioside A	11.2 ± 0.8	2.9–25.0

**Table 7:** Mean energy intake and macronutrient intakes (in percentages of energy intake) presented as mean and standard error.

	All (*n* = 56)
	Mean ± SEM
Total energy (kJ)	10,800.2 ± 692.9
Total fat (%)	35.1 ± 1.9
Protein (%)	20.1 ± 0.8
CHO (%)	44.9 ± 1.9
Sugar (%)	13.6 ± 0.8
Starch (%)	26.1 ± 1.2
Fibre (%)	4.3 ± 0.2

**Table 8:** Consumption of added-sugar and specific sugar-sweetened foods in grams ^a,b,c^.

	All (*n* = 56)
	Grams/Day
Sugar ^b^	9.2 ± 2.2
Sweet biscuits	8.2 ± 1.4
Cakes	10.1 ± 1.6
Flavoured milk	1.7 ± 0.3
Fruit spreads (jam)	2.8 ± 0.6
Ice-cream	12.1 ± 2.5
Chocolate	16.6 ± 2.2
Bread	95.3 ± 6.3
Breakfast cereals ^c^	21.0 ± 3.3

^a^ All values are presented as the mean ± SEM; ^b^ Sugar: calculated from teaspoons of sugar used per day; ^c^ Breakfast cereals: includes all bran, branflakes, weet-bix, cornflakes, porridge and muesli.

## Abbreviations

The following abbreviations are used in this manuscript:

BMI	Body mass index
DT	Detection threshold
RT	Recognition threshold
ST	Suprathreshold
gLMS	General Labeled Magnitude Scale
HIS	High intensity sweetener
FFQ	Food frequency questionnaire
SEM	Standard error of mean
SD	Standard deviation
ANOVA	Analysis of variance

## References

[1] Swinburn B.A., Sacks G., Hall K.D., McPherson K., Finegood D.T., Moodie M.L., Gortmaker S.L. (2011). The global obesity pandemic: Shaped by global drivers and local environments. Lancet.

[2] Bellisle F., Drewnowski A. (2007). Intense sweeteners, energy intake and the control of body weight. Eur. J. Clin. Nutr..

[3] Bermudez O.I., Gao X. (2010). Greater consumption of sweetened beverages and added sugars is associated with obesity among us young adults. Ann. Nutr. Metab..

[4] Malik V.S., Schulze M.B., Hu F.B. (2006). Intake of sugar-sweetened beverages and weight gain: A systematic review. Am. J. Clin. Nutr..

[5] Malik V.S., Pan A., Willett W.C., Hu F.B. (2013). Sugar-sweetened beverages and weight gain in children and adults: A systematic review and meta-analysis. Am. J. Clin. Nutr..

[6] Lim S.S., Vos T., Flaxman A.D., Danaei G., Shibuya K., Adair-Rohani H., AlMazroa M.A., Amann M., Anderson H.R., Andrews K.G. (2012). A comparative risk assessment of burden of disease and injury attributable to 67 risk factors and risk factor clusters in 21 regions, 1990–2010: A systematic analysis for the global burden of disease study 2010. Lancet.

[7] Finucane M.M., Stevens G.A., Cowan M.J., Danaei G., Lin J.K., Paciorek C.J., Singh G.M., Gutierrez H.R., Lu Y., Bahalim A.N. (2011). National, regional, and global trends in body-mass index since 1980: Systematic analysis of health examination surveys and epidemiological studies with 960 country-years and 9.1 million participants. Lancet.

[8] Kelly T., Yang W., Chen C., Reynolds K., He J. (2008). Global burden of obesity in 2005 and projections to 2030. Int. J. Obes..

[9] Breslin P.A. (2013). An evolutionary perspective on food and human taste. Curr. Biol..

[10] Simon S.A., De Araujo I.E., Gutierrez R., Nicolelis M.A. (2006). The neural mechanisms of gustation: A distributed processing code. Nat. Rev. Neurosci..

[11] Bellisle F. (2015). Intense sweeteners, appetite for the sweet taste, and relationship to weight management. Curr. Obes. Rep..

[12] Zheng M., Allman-Farinelli M., Heitmann B.L., Rangan A. (2015). Substitution of sugar-sweetened beverages with other beverage alternatives: A review of long-term health outcomes. J. Acad. Nutr. Diet..

[13] Keast R.S. (2016). Effects of sugar and fat consumption on sweet and fat taste. Curr. Opin. Behav. Sci..

[14] Pangborn R.M., Pecore S.D. (1982). Taste perception of sodium chloride in relation to dietary intake of salt. Am. J. Clin. Nutr..

[15] Keller K.L., Steinmann L., Nurse R.J., Tepper B.J. (2002). Genetic taste sensitivity to 6-n-propylthiouracil influences food preference and reported intake in preschool children. Appetite.

[16] Drewnowski A., Henderson S.A., Levine A., Hann C. (1999). Taste and food preferences as predictors of dietary practices in young women. Public Health Nutr..

[17] Shepherd R., Farleigh C., Land D. (1984). Preference and sensitivity to salt taste as determinants of salt-intake. Appetite.

[18] Yackinous C.A., Guinard J.-X. (2002). Relation between prop (6-n-propylthiouracil) taster status, taste anatomy and dietary intake measures for young men and women. Appetite.

[19] Stewart J.E., Feinle-Bisset C., Golding M., Delahunty C., Clifton P.M., Keast R.S. (2010). Oral sensitivity to fatty acids, food consumption and bmi in human subjects. Br. J. Nutr..

[20] Stewart J.E., Feinle-Bisset C., Keast R.S. (2011). Fatty acid detection during food consumption and digestion: Associations with ingestive behavior and obesity. Prog. Lipid Res..

[21] Cicerale S., Riddell L.J., Keast R.S. (2012). The association between perceived sweetness intensity and dietary intake in young adults. J. Food Sci..

[22] Sartor F., Donaldson L.F., Markland D.A., Loveday H., Jackson M.J., Kubis H.-P. (2011). Taste perception and implicit attitude toward sweet related to body mass index and soft drink supplementation. Appetite.

[23] Tucker R.M., Edlinger C., Craig B.A., Mattes R.D. (2014). Associations between bmi and fat taste sensitivity in humans. Chem. Senses.

[24] Umabiki M., Tsuzaki K., Kotani K., Nagai N., Sano Y., Matsuoka Y., Kitaoka K., Okami Y., Sakane N., Higashi A. (2010). The improvement of sweet taste sensitivity with decrease in serum leptin levels during weight loss in obese females. Tohoku J. Exp. Med..

[25] Grinker J., Hirsch J., Smith D.V. (1972). Taste sensitivity and susceptibility to external influence in obese and normal weight subjects. J. Personal. Soc. Psychol..

[26] Malcolm R., O’Neil P., Hirsch A., Currey H., Moskowitz G. (1979). Taste hedonics and thresholds in obesity. Int. J. Obes..

[27] Navabi N., Hamzeh F. (2008). Investigation of the relationship between sweet taste sensitivity and body mass index. Daneshvar Med..

[28] Frijters J.E., Rasmussen-Conrad E.L. (1982). Sensory discrimination, intensity perception, and affective judgment of sucrose-sweetness in the overweight. J. Gen. Psychol..

[29] Wooley O.W., Wooley S.C., Dunham R.B. (1972). Calories and sweet taste: Effects on sucrose preference in the obese and nonobese. Physiol. Behav..

[30] Rodin J., Moskowitz H.R., Bray G.A. (1976). Relationship between obesity, weight loss, and taste responsiveness. Physiol. Behav..

[31] Rodin J. (1975). Effects of obesity and set point on taste responsiveness and ingestion in humans. J. Comp. Physiol. Psychol..

[32] Thompson D.A., Moskowitz H.R., Campbell R.G. (1977). Taste and olfaction in human obesity. Physiol. Behav..

[33] Simone M., Pangborn R. (1957). Consumer acceptance methodology-one *vs*. 2 samples. Food Technol..

[34] Thompson D.A., Moskowitz H.R., Campbell R.G. (1976). Effects of body weight and food intake on pleasantness ratings for a sweet stimulus. J. Appl. Physiol..

[35] Witherly S., Pangborn R.M., Stern J.S. (1980). Gustatory responses and eating duration of obese and lean adults. Appetite.

[36] Drewnowski A., Kurth C.L., Rahaim J.E. (1991). Taste preferences in human obesity: Environmental and familial factors. Am. J. Clin. Nutr..

[37] Sørensen L.B., Møller P., Flint A., Martens M., Raben A. (2003). Effect of sensory perception of foods on appetite and food intake: A review of studies on humans. Int. J. Obes..

[38] Bartoshuk L.M., Duffy V.B., Hayes J.E., Moskowitz H.R., Snyder D.J. (2006). Psychophysics of sweet and fat perception in obesity: Problems, solutions and new perspectives. Philos. Trans. R. Soc. B Biol. Sci..

[39] Webb J., Bolhuis D.P., Cicerale S., Hayes J.E., Keast R. (2015). The relationships between common measurements of taste function. Chemosens. Percept..

[40] Keast R.S., Roper J. (2007). A complex relationship among chemical concentration, detection threshold, and suprathreshold intensity of bitter compounds. Chem. Senses.

[41] Bartoshuk L.M. (2000). Comparing sensory experiences across individuals: Recent psychophysical advances illuminate genetic variation in taste perception. Chem. Senses.

[42] International Organisation for Standardization Sensory Analysis—Methodology—Method of Investigating Sensitivity of Taste. http://www.iso.org/iso/iso_catalogue/catalogue_ics/catalogue_detail_ics.htm?csnumber=9636.

[43] Keast R.S., Canty T.M., Breslin P.A. (2004). Oral zinc sulfate solutions inhibit sweet taste perception. Chem. Senses.

[44] Meilgaard M., Civille G., Carr B. (2007). Overall difference tests: Does a sensory difference exist between samples. Sens. Eval. Tech..

[45] Wise P.M., Breslin P.A. (2013). Individual differences in sour and salt sensitivity: Detection and quality recognition thresholds for citric acid and sodium chloride. Chem. Senses.

[46] Delwiche J.F., Buletic Z., Breslin P.A. (2001). Covariation in individuals’ sensitivities to bitter compounds: Evidence supporting multiple receptor/transduction mechanisms. Percept. Psychophys..

[47] World Health Organization (2004). International Statistical Classification of Diseases and Related Health Problems.

[48] Australian Health Foundation Waist Measurement. http://heartfoundation.org.au/your-heart/know-your-risks/healthy-weight/waist-measurement2016.

[49] Hodge A., Patterson A.J., Brown W.J., Ireland P., Giles G. (2000). The anti cancer council of victoria ffq: Relative validity of nutrient intakes compared with weighed food records in young to middle-aged women in a study of iron supplementation. Aust. N. Z. J. Public Health.

[50] Mahar A., Duizer L. (2007). The effect of frequency of consumption of artificial sweeteners on sweetness liking by women. J. Food Sci..

[51] Barros R., Moreira A., Fonseca J., Ferraz de Oliveira J., Delgado L., Castel-Branco M., Haahtela T., Lopes C., Moreira P. (2008). Adherence to the mediterranean diet and fresh fruit intake are associated with improved asthma control. Allergy.

[52] Perneger T.V. (1998). What’s wrong with Bonferroni adjustments. Br. Med. J..

[53] Armstrong R.A. (2014). When to use the Bonferroni correction. Ophthalmic Physiol. Opt..

[54] Wise P.M., Nattress L., Flammer L.J., Beauchamp G.K. (2016). Reduced dietary intake of simple sugars alters perceived sweetness taste intensity but not perceived pleasantness. Am. J. Clin. Nutr..

[55] Bartoshuk L.M. (1978). The psychophysics of taste. Am. J. Clin. Nutr..

[56] Duffy V.B., Peterson J.M., Bartoshuk L.M. (2004). Associations between taste genetics, oral sensation and alcohol intake. Physiol. Behav..

[57] Mojet J., Heidema J., Christ-Hazelhof E. (2004). Effect of Concentration on Taste-Taste Interactions in Foods for Elderly and Young Subjects. Chem. Senses.

[58] Drewnowski A., Greenwood M. (1983). Cream and sugar: Human preferences for high-fat foods. Physiol. Behav..

[59] Beydoun M., Wang Y. (2008). How do socio-economic status, perceived economic barriers and nutritional benefits affect quality of dietary intake among us adults?. Eur. J. Clin. Nutr..

[60] Vereecken C.A., Inchley J., Subramanian S., Hublet A., Maes L. (2005). The relative influence of individual and contextual socio-economic status on consumption of fruit and soft drinks among adolescents in Europe. Eur. J. Public Health.

[61] Hulshof K., Brussaard J., Kruizinga A., Telman J., Löwik M. (2003). Socio-economic status, dietary intake and 10 y trends: The dutch national food consumption survey. Eur. J. Clin. Nutr..

[62] Simchen U., Koebnick C., Hoyer S., Issanchou S., Zunft H.-J. (2006). Odour and taste sensitivity is associated with body weight and extent of misreporting of body weight. Eur. J. Clin. Nutr..

[63] Donaldson L.F., Bennett L., Baic S., Melichar J.K. (2009). Taste and weight: Is there a link?. Am. J. Clin. Nutr..

[64] Drewnowski A., Brunzell J.D., Sande K., Iverius P., Greenwood M. (1985). Sweet tooth reconsidered: Taste responsiveness in human obesity. Physiol. Behav..

[65] Drewnowski A. (1998). Energy density, palatability, and satiety: Implications for weight control. Nutr. Rev..

